# Supplementary material for: Gate-tunable large magnetoresistance in an all-semiconductor spin valve device
Source: Nat Commun. 2017 Nov 27;8:1807. doi: 10.1038/s41467-017-01933-2 (PMC5702618; doi:10.1038/s41467-017-01933-2)
Supplement: Supplementary file 1 — Supplementary Information [file 41467_2017_1933_MOESM1_ESM.pdf]

Supplementary Note 1. Device characterization.

In this Note, we present the results of basic characterization measurements of the non-gated spin injection device, used for the experiments summarized in Figures 2 and 3.

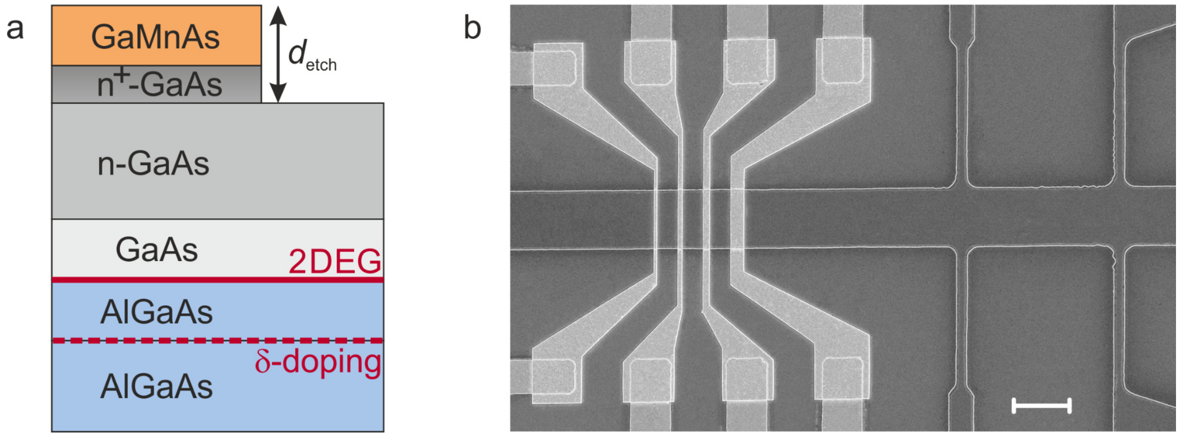

Supplementary Figure 1 | Wafer design and device layout. (a) The layer sequence of the wafer used to fabricate the investigated devices. d_{etch} corresponds to the thickness of the material etched away to confine the electric transport within the 2DES (see Ref. 1 and Methods). **(b)** SEM picture of a typical non-gated device. It consists of a 10 μm wide channel with four ferromagnetic contacts (bright areas) on top. Four potential probes can be seen to the right of the magnetic contacts. Contacts to the mesa and to the voltage probes are not shown. The scale bar corresponds to 10 μm.

Supplementary Figure 1 shows the layer sequence of the wafer used to fabricate devices (see Methods) and the SEM picture of one of our devices without a gate. The transport channel containing the 2DES is contacted by (Ga,Mn)As/GaAs Esaki diodes covered with an Au/Ti metal film which connects the degenerately p-doped (Ga,Mn)As to the contact pads, seen in the SEM picture. The widths of the FM electrodes are 500 nm, 700 nm, 1 μm and 2 μm, separated by (center to center) 3.6 μm, 3.85 μm and 4.5 μm, respectively. The two narrowest electrodes were used as source (S) and drain (D) in the two-terminal experiments described in the main text. The four potential probes seen to the right of FM contacts were used to determine charge transport parameters in magnetotransport measurements (see Supplementary Figure 3). Six large 100 μm × 100 μm contacts are used to provide electrical contact to the channel: two at each end of the mesa and four at the voltage probes. They also consist of Esaki diodes topped with an Au/Ti layer. These contacts, far away (in terms of the spin diffusion length) from the spin-injector/detector contacts serve as current/voltage probes in magnetotransport measurements (Supplementary Figure 3) and as reference contacts in nonlocal spin injection experiments (Supplementary Figure 4).

Supplementary Figure 2 shows current-voltage (I-V) characteristics of S and D contacts. These traces are typical for our devices[1,2] employing Esaki tunnel diodes. They are nearly linear in the low-bias region up to $V_{3T} \cong \pm 0.25$ V. Here, V_{3T} is the three-terminal voltage,

measured as sketched in the inset of Supplementary Figure 2. Between $0.25\ \mathrm{V} \lesssim V_{3\mathrm{T}} \lesssim 0.6\ \mathrm{V}$ the non-linear region of a negative differential resistance (NDR) prevails.

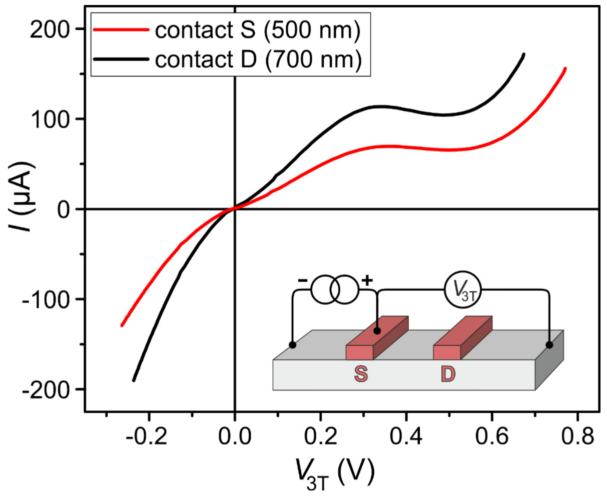

Supplementary Figure 2 | I-V characteristics. *I-V* characteristics of the two narrow FM electrodes, used as source and drain leads. $V_{3\mathrm{T}}$ is the voltage drop across the interface, sketched in the inset. Zero-bias resistance area product is $R_{\mathrm{ZB}}A \approx 2.5 \pm 0.3 \times 10^{-8}\ \Omega\mathrm{m}^2$

Supplementary Figure 3 shows magnetotransport measurements performed in an out-of-plane magnetic field B. Shown are Hall resistivity ρ_{xy} and longitudinal resistivity ρ_{xx} . From the measurements we extract the sheet resistance $R_{\mathrm{s}} = 87\ \Omega$, carrier density $n_{\mathrm{s}} = 2.5 \times 10^{11}\ \mathrm{cm}^2$, carrier mobility $\mu_{\mathrm{e}} = 3.1 \times 10^5\ \mathrm{cm}^2/\mathrm{Vs}$, and mean free path $l_{\mathrm{e}} = 2.5\ \mu\mathrm{m}$.

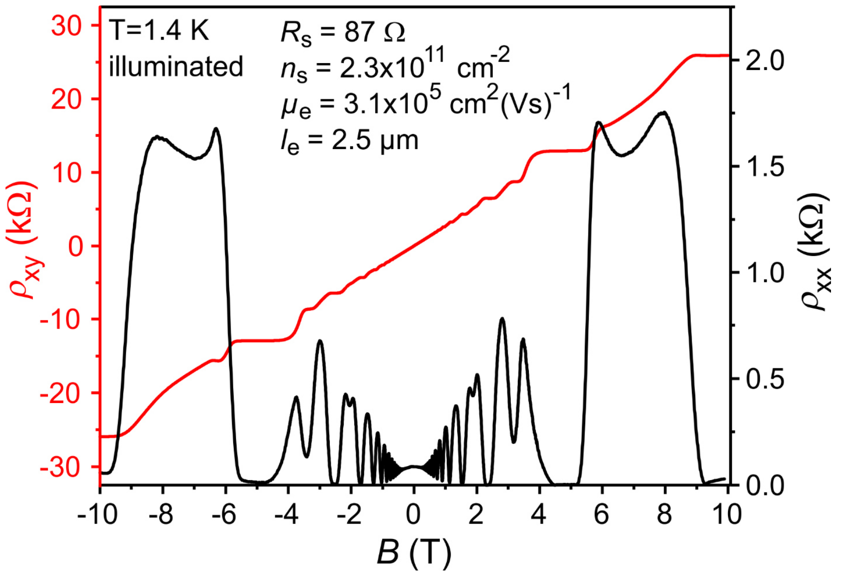

Supplementary Figure 3 | Magnetotransport experiment in an out-of-plain magnetic field. Hall resistivity (ρ_{xy}) and longitudinal resistivity (ρ_{xx}) are measured at the voltage probes shown in Supplementary Figure 1b. The resistivity ρ_{xx} vanishes at integer filling factors indicating that no parallel transport channel exists.

Supplementary Figure 4 summarizes the results of nonlocal spin valve (NLSV) measurements performed with low AC excitation current, in the configuration as shown in

Supplementary Figure 4b. Here, the 500 nm wide FM electrode serves as injector and the other contacts as nonlocal detectors. Spin accumulation generated at the injector diffuses along the channel and is measured by detectors as nonlocal voltage V^{nl}.[3] Sweeping B along the long axis of the FM contacts from +0.5 T to –0.5 T and back switches the detected V^{nl} (Supplementary Figure 4a) whenever the magnetization orientation of injector and detector switches from parallel (P) to anti-parallel (AP) and vice versa. The amplitude of the NLSV signal, i.e. the change of the nonlocal voltage ΔV^{nl}, is, in the tunneling regime of spin injection, given by[4]

$$\Delta V^{\mathrm{nl}} = \frac{P_{\mathrm{inj}} P_{\mathrm{det}} I R_{\mathrm{s}} \lambda_{\mathrm{s}}}{W} \exp(-\frac{d}{\lambda_{\mathrm{s}}}),$$

where $P_{\mathrm{inj(det)}}$ is the spin injection (detection) efficiency, W the width of the channel, R_{s} the sheet resistance, λ_{s} the spin diffusion length and d the injector-detector separation for a given pair of contacts.

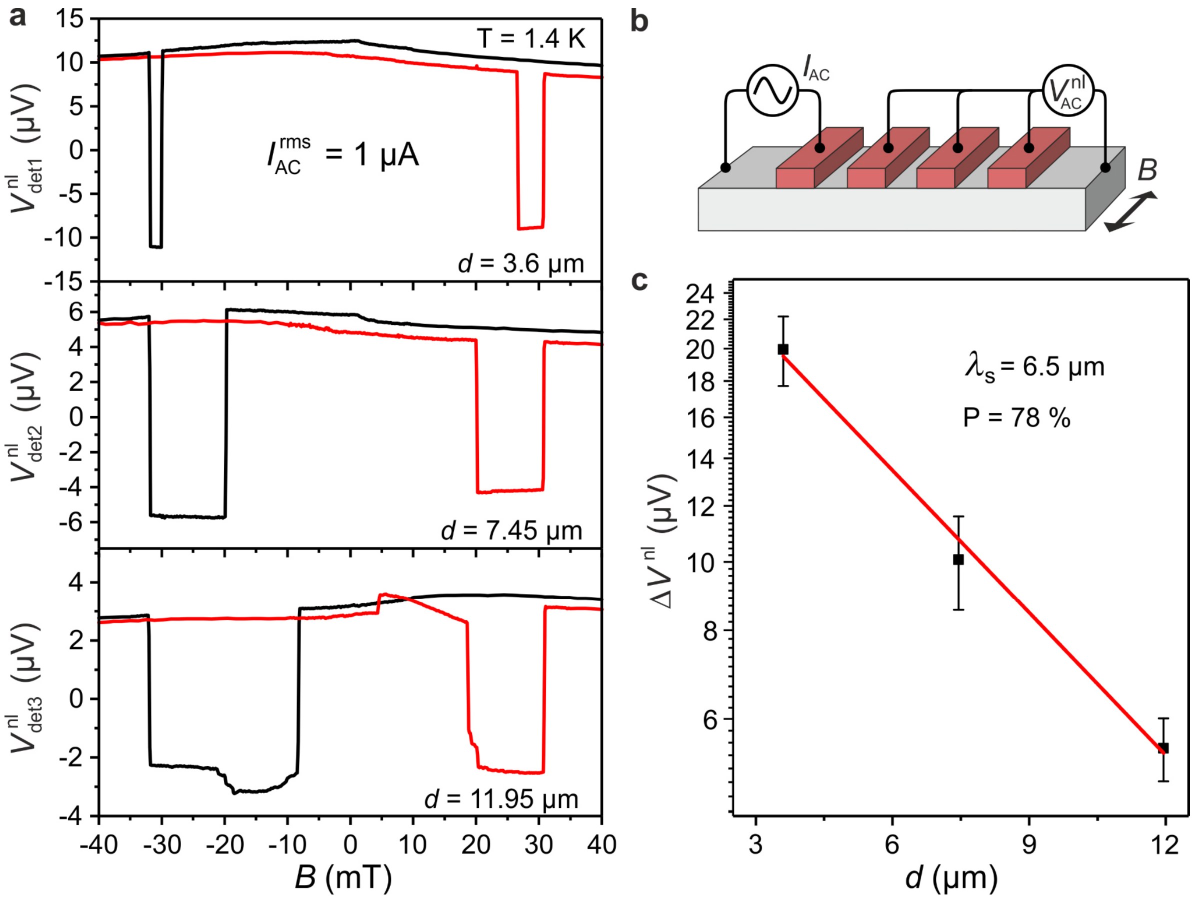

Supplementary Figure 4 | Nonlocal spin valve experiments. (a) Nonlocal spin valve experiments measured at three distances d from the injector using the configuration sketched in **(b)**. Red (black) traces correspond to sweeping B in a positive (negative) direction. The AC (f = 13 Hz) excitation current was $I_{\mathrm{AC}}^{\mathrm{rms}} = 1\ \mu\mathrm{A}$ **(c)** Distance dependence of ΔV^{nl}, used to extract the spin diffusion length $\lambda_{\mathrm{s}} = 6.5\ \mu m$ and spin injection efficiency of $P = 78\ \%$. Error bars indicate the uncertainty when ΔV^{nl} is determined form the experimental traces in **(a)**.

From the distance dependence $\Delta V^{\mathrm{nl}}(d)$ (Supplementary Figure 4c) we extract the spin diffusion length $\lambda_{\mathrm{s}} = 6.5$ µm, and the spin injection efficiency P=78%, assuming $P_{\mathrm{inj}} \cong P_{\mathrm{det}} = P$ for low bias values. Similar results were also obtained from low excitation DC measurements.

From the extracted charge transport and spin transport parameters we calculate the channel spin resistance as $R_{\mathrm{ch}} = \rho\lambda_{\mathrm{s}}\frac{1}{Wt} = R_{\mathrm{s}}\lambda_{\mathrm{s}}\frac{1}{W} \approx 57\ \Omega$, with the channel cross-section $A = Wt$, where $W = 10$ µm is the width and t is the thickness of the 2DES channel.[5] We estimate the spin resistance of the tunnel contact as $R_{\mathrm{T}} = \frac{R_{\mathrm{ZB}}}{1-P^2} \approx 14.5$ kΩ. Both parameters determine the conditions for efficient spin injection and detection in a given system. According to the standard model, the maximum spin signal is observed when $R_{\mathrm{ch}} \ll R_{\mathrm{T}} \ll R_{\mathrm{ch}}\,\lambda_{\mathrm{s}}/d$.[5,6] The left condition is clearly satisfied for our device, indicating that the device works in the tunneling regime, enabling efficient spin injection. The right condition is not fulfilled, as the ratio $R_{\mathrm{T}}/R_{\mathrm{ch}} \approx 250$ is two orders of magnitude too large. This means that the dwell time of electrons in the channel is much larger than the spin relaxation time.[4–7] These values are typical for all our devices and a large $R_{\mathrm{T}}/R_{\mathrm{ch}}$ ratio is typical for semiconductor-based spin devices. As a high interface resistance indicates also larger spin-independent contribution to the two-terminal resistance $R^{\mathrm{P(AP)}}$, it results in a low magnetoresistance ratio MR = $\Delta R/R^{\mathrm{P}}$.[8–13]

Supplementary Note 2. Magnetoresistance of the gated device.

In this Note, we present additional two-terminal local measurements on the gated sample, used for the gate-controlled experiments presented in Figure 4. The *I-V* characteristic measured between source and drain, shown in Supplementary Figure 5a, is almost point symmetric with respect to the origin. Spin valve signals are shown for two bias currents in Supplementary Figures 5b and d. The MR, i.e., $\Delta R/R^{\mathrm{P}}$ with the resistance difference ΔR between parallel and anti-parallel orientation, is shown in Supplementary Figure 5c as a function of bias current and reaches values of up to 80%.

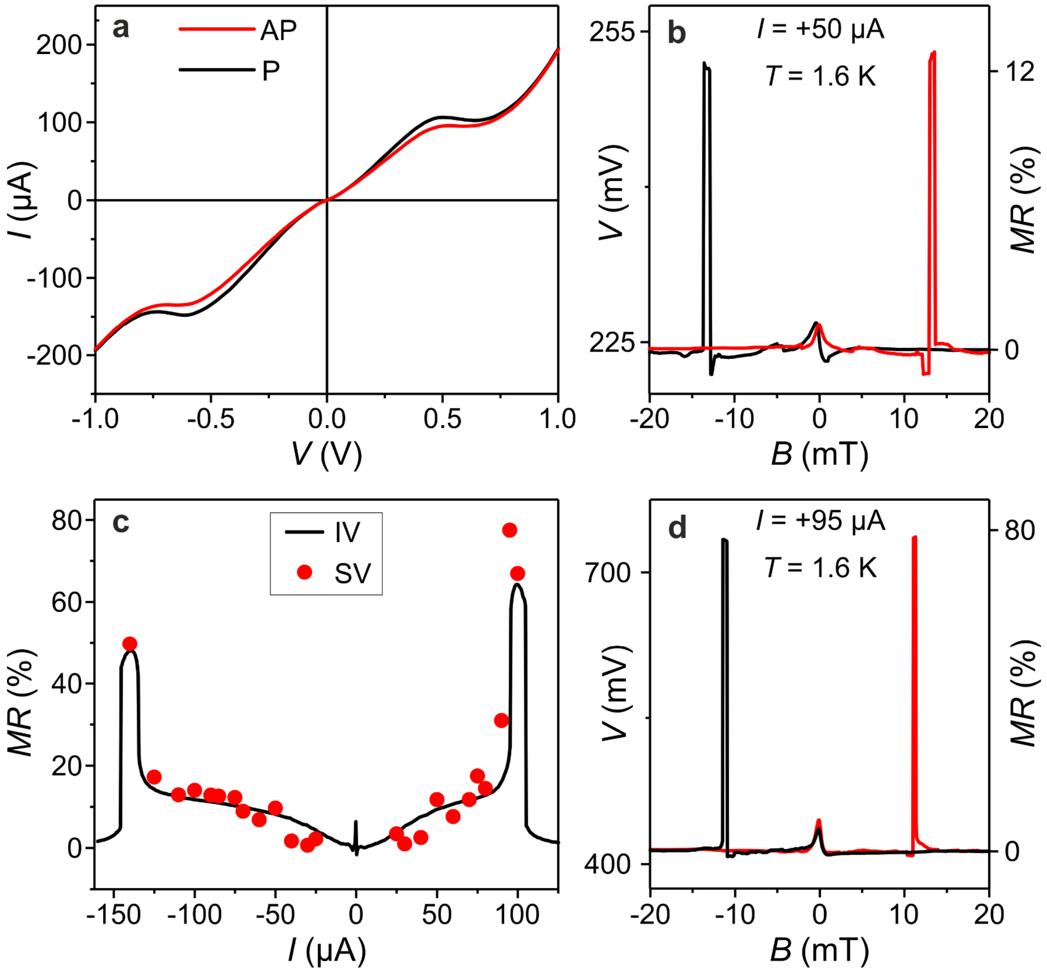

Supplementary Figure 5 | Magnetoresistance of the gated device. (a) Two-terminal *I–V* curves of the gated device in the open configuration, with V_G=+1.5 V. **(b)**,**(d)** 2T spin valve measurements in the linear **(b)** and the non-linear regime **(d)**. Red (black) traces correspond to sweeping *B* in a positive (negative) direction. **(c)** Bias dependence of the magnetoresistance ratio MR. Red circles indicate data points from spin valve measurements and the line represents data obtained by subtracting *I–V* curves obtained in the parallel and antiparallel configuration for each current.

Supplementary Note 3. Influence of an electric field on the spin signal.

In this Note, we explain in detail how we constructed Figure 3. There we compared the measured 2T local signal ΔR with values $\Delta R^{E=0}$ expected from standard theory without electric field effects, and with $\Delta R^{E\neq0}$, for which electric field's effects have been included. As an example, we discuss the procedure for an injection current of 52 μA.

Supplementary Figure 6a displays the results of 2T local spin valve measurements with a positively biased source (S) contact. We measure a large SV signal $\Delta V = 60$ mV, which we plot in Fig. 3 (black triangles) as resistance $\Delta R = \frac{\Delta V}{I} = 1.1$ kΩ. This value we compare with $\Delta V^{E=0} = \Delta V_{\mathrm{S,D}}^{E=0} + \Delta V_{\mathrm{D,S}}^{E=0}$, where $\Delta V_{\mathrm{S,D}}^{E=0}$ and $\Delta V_{\mathrm{D,S}}^{E=0}$ correspond to, respectively, the voltage change at the source due to the spin accumulation generated at the drain and the other way around. Both $\Delta V_{\mathrm{S,D}}^{E=0} = \Delta V_{\mathrm{S,D}}^{\mathrm{nl}}$ and $\Delta V_{\mathrm{D,S}}^{E=0} = \Delta V_{\mathrm{D,S}}^{\mathrm{nl}}$ are measured in nonlocal experiments shown in Supplementary Figures 6b and 6c, respectively. From these experiments we get $\Delta V_{\mathrm{S,D}}^{\mathrm{nl}} = 0.83$ mV and $\Delta V_{\mathrm{D,S}}^{\mathrm{nl}} = 1.02$ mV, resulting in $\Delta V^{E=0} = \Delta V_{\mathrm{S,D}}^{\mathrm{nl}} + \Delta V_{\mathrm{D,S}}^{\mathrm{nl}} = 1.85$ mV. This and corresponding values taken at other bias currents are shown in Fig. 3 (blue squares) as resistance $\Delta R^{E=0} = \frac{\Delta V^{E=0}}{I}$. Next, we show how to estimate $\Delta V^{E\neq0}$. We first decompose the signal as $\Delta V^{E\neq0} = \eta_{\mathrm{S}}\Delta V_{\mathrm{S,D}}^{\mathrm{nl}} + \eta_{\mathrm{D}}\Delta V_{\mathrm{D,S}}^{\mathrm{nl}}$, where $\eta_{\mathrm{S(D)}}$ is a factor representing the effect of the electric field on the signal component detected at the source (drain). We write this factor as $\eta_{\mathrm{S}} = \eta_{\mathrm{S}}^{(\mathrm{i})}\eta_{\mathrm{S}}^{(\mathrm{ii})}$, where $\eta_{\mathrm{S}}^{(\mathrm{i})}$ describes the effect of drift on spin transport in the channel[14] and $\eta_{\mathrm{S}}^{(\mathrm{ii})}$ the effect of bias on the spin-to-charge conversion of the biased FM contact.[15,16]

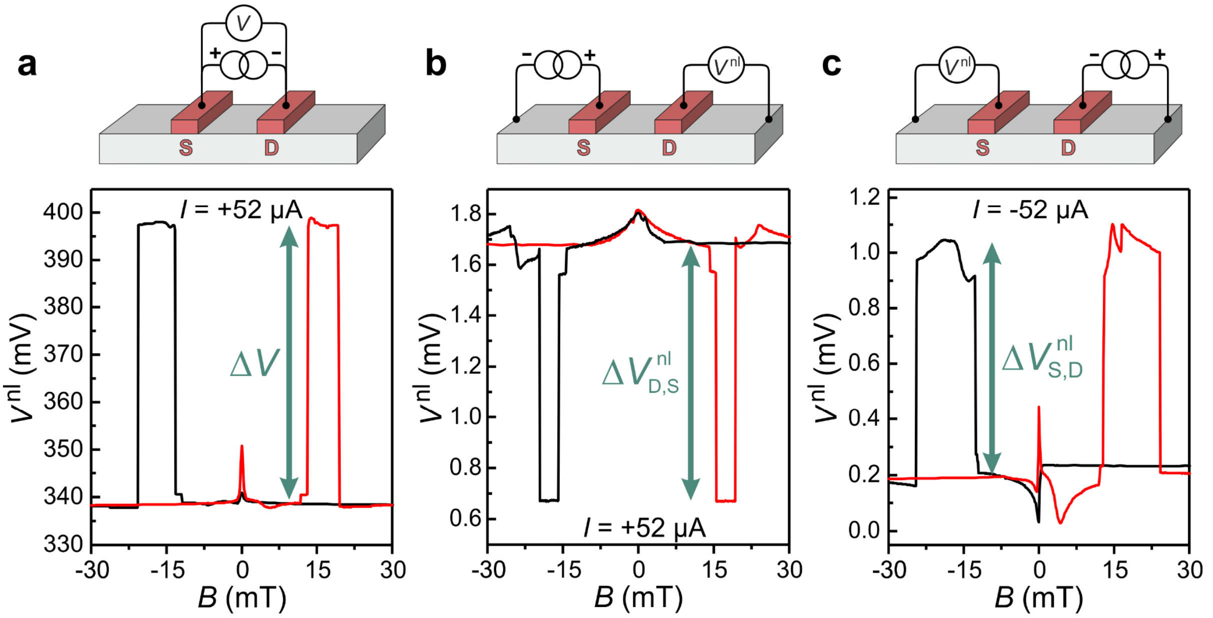

Supplementary Figure 6 | Comparison of local and nonlocal SV signals. Two-terminal local (2TL) and nonlocal (NL) spin valve measurements for an injection current of 52 μA. **(a)** 2TL measurements with a positive bias on the source and a negative bias on the drain. **(b)** NL measurements with a positively biased S as an injector and the non-biased D as a detector. **(c)** NL measurements with a negatively biased D as an injector and the non-biased S as a detector.

Estimate of $\eta_{\mathrm{S}}^{(\mathrm{i})}$ and $\eta_{\mathrm{D}}^{(\mathrm{i})}$: Drift in the channel influences the diffusion of spins along the channel, which in the absence of drift is described by the intrinsic spin diffusion length λ_{s}. The presence of an electric field enhances diffusion of spins in drift direction ("down-stream") and suppresses diffusion of spins in the opposite direction ("up-stream"). This can be described for both cases by a modified spin diffusion lengths, given by[14,17] $\lambda_{\mathrm{s,d(u)}} = \left[-(+)\frac{|E|}{2}\frac{\mu_{\mathrm{e}}}{D_{\mathrm{s}}} + \sqrt{\left(\frac{|E|}{2}\frac{\mu_{\mathrm{e}}}{D_{\mathrm{s}}}\right)^2 + \frac{1}{\lambda_{\mathrm{s}}^2}}\right]^{-1}$, where λ_{s} and $\lambda_{\mathrm{s,d(u)}}$ are the intrinsic and down- (up-) stream spin diffusion lengths in the channel, respectively, μ_{e} is the electron mobility, D_{s} is the spin diffusivity and E the electric field. For 2T experiments shown in Supplementary Figure 6a, i.e. with positively biased source and negatively biased drain, the drift carries electrons from drain towards source. Therefore, diffusion of spin accumulation from drain towards source is enhanced by drift and described by $\lambda_{\mathrm{s,d}}$. The component of the spin valve signal $\Delta V_{\mathrm{S,D}}$ due to spin accumulation generated at the drain and detected at the source is then enhanced by drift, with an enhancement factor defined as $\eta_{\mathrm{S}}^{(\mathrm{i})} = \frac{\Delta V_{\mathrm{S,D}}^{E\neq 0}}{\Delta V_{\mathrm{S,D}}^{E=0}} = \frac{\exp(-\frac{d}{\lambda_{\mathrm{s,d}}})}{\exp(-\frac{d}{\lambda_{\mathrm{s}}})}$. Correspondingly, diffusion of spin accumulation from source towards drain is suppressed by drift and described by $\lambda_{s,u}$. The component of the

spin valve signal $\Delta V_{D,S}$ due to spin accumulation generated at the source and detected at the drain is suppressed by drift, with a suppression factor defined as $\eta_{\mathrm{D}}^{(\mathrm{i})} = \frac{\Delta V_{\mathrm{D,S}}^{E\neq 0}}{\Delta V_{\mathrm{D,S}}^{E=0}} = \frac{\exp(-\frac{d}{\lambda_{\mathrm{s,u}}})}{\exp(-\frac{d}{\lambda_{\mathrm{s}}})}$.

We now exemplarily calculate the factors $\eta_{\mathrm{S}}^{(\mathrm{i})}$ and $\eta_{\mathrm{D}}^{(\mathrm{i})}$ for I = +52 μA. The corresponding electric field in the channel is $E = \frac{IR_{\mathrm{s}}}{W} = 4.21$ V/cm ($W = 10\ \mu$m is the channel width). $\lambda_{\mathrm{s}} = 6.5\ \mu$m and electron mobility μ_{e} are taken from measurements described in Supplementary Note 1. With the spin diffusivity $D_{\mathrm{s}} = \lambda_{\mathrm{s}}^2/\tau_{\mathrm{s}}$, where the spin relaxation time $\tau_{\mathrm{s}} \approx 1$ ns has been measured by nonlocal Hanle spin precession[18,19] we obtain $\lambda_{\mathrm{s,d}}(I = 52\ \mu\mathrm{A}) = 16\ \mu\mathrm{m}$ and $\lambda_{\mathrm{s,u}}(I = 52\ \mu\mathrm{A}) = 2.66\ \mu\mathrm{m}$ from the expression given above. For the source–drain separation of 3.6 μm this gives the corresponding enhancement and suppression factors $\eta_{\mathrm{S}}^{(\mathrm{i})}(I = 52\ \mu A) \cong 1.39$ and $\eta_{\mathrm{D}}^{(\mathrm{i})}(I = 52\ \mu A) \cong 0.45$.

The effect of biased FM electrode on the measured spin signal, i.e. $\eta_{\mathrm{S}}^{(\mathrm{ii})}$ and $\eta_{\mathrm{D}}^{(\mathrm{ii})}$, we extract from the nonlocal experiments shown in Supplementary Figures 7 and 8, respectively. Supplementary Figure 7a shows the nonlocal spin valve signal detected at the source contact with an AC injection current $I_{\mathrm{AC}}^{\mathrm{rms}} = 700$ nA flowing through the drain. The signal amplitude is $\Delta V_{\mathrm{S,D}}^{\mathrm{nl}} = 7.65$ μV. Supplementary Figure 7b displays the NLSV obtained when an additional DC current of $I_{\mathrm{DC}}^{\mathrm{S}} = +52$ μA is applied to the source, i.e. to the nonlocal detector. Note that both amplitude and polarity of the applied DC current match the magnitude and the polarity of the current flowing through the source in the 2T measurements, shown in Supplementary Figure 6a. The biased detector exhibits a much larger amplitude of the signal $\Delta V_{\mathrm{S,D}}^{\mathrm{nl},E\neq 0}\left(I_{\mathrm{DC}}^{\mathrm{S}} = +52\ \mu\mathrm{A}\right) = 337$ μV compared to the non-biased case of $\Delta V_{\mathrm{S,D}}^{\mathrm{nl}}\left(I_{\mathrm{DC}}^{\mathrm{S}} = 0\right) = 7.65$ μV. From both values we extract

$$\eta_{\mathrm{S}}^{(\mathrm{ii})} = \Delta V_{\mathrm{S,D}}^{\mathrm{nl},E\neq 0}\left(I_{\mathrm{DC}}^{\mathrm{S}} = +52\ \mu\mathrm{A}\right)/\Delta V_{\mathrm{S,D}}^{\mathrm{nl}}\left(I_{\mathrm{DC}}^{\mathrm{S}} = 0\right) = 44.5,$$

i.e., biasing the source in positive direction enhances the signal detected at this contact by a factor of 44.5 for $I_{\mathrm{DC}}^{\mathrm{S}} = +52$ μA.

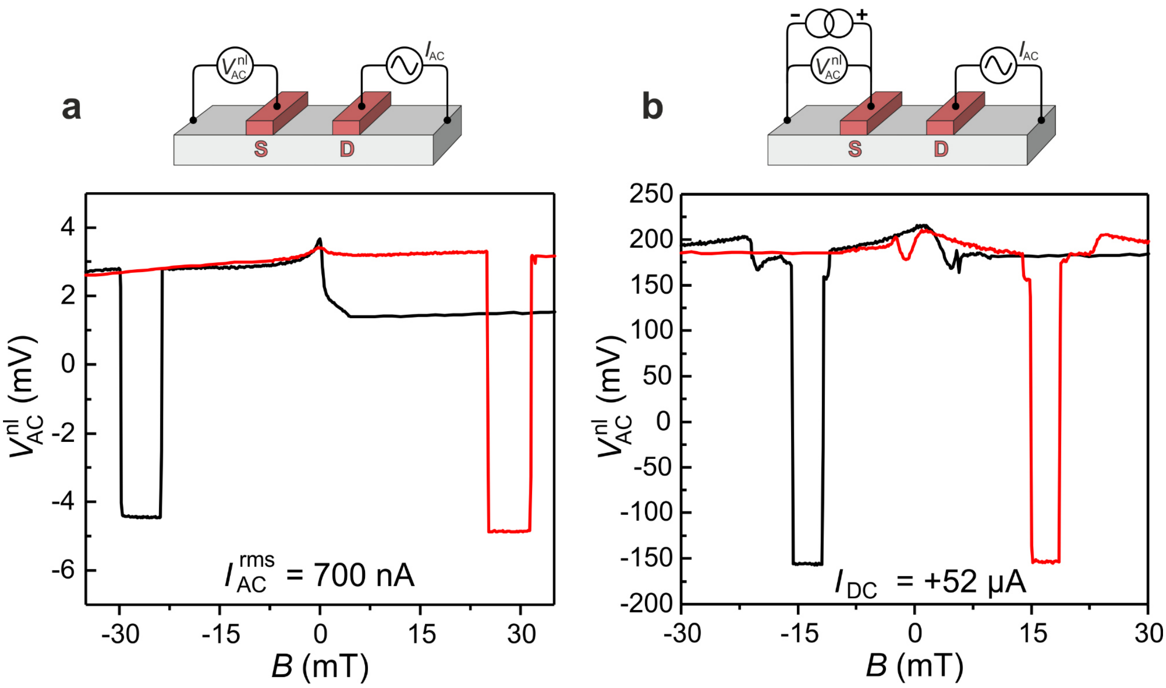

Supplementary Figure 7 | Effect of forward bias on the nonlocal spin valve signal. Measurements performed in order to determine the enhancement of the spin-related voltage detected at the source, as a result of biasing the lead in the forward direction. **(a)** Nonlocal spin valve signal detected at the non-biased source contact with an AC injection current applied to the drain FM. **(b)** Same measurement configuration as in **(a)** with an additional DC current applied to the nonlocal detector (S).

Supplementary Figure 8 shows analogous measurements with source as an injector and drain as a nonlocal detector for both non-biased drain (Supplementary Fig. 8a) and drain biased with a current of $I_{\mathrm{DC}} = -52\ \mu\mathrm{A}$ (Supplementary Fig. 8b). The negative bias applied to the drain suppresses somewhat the measured spin signal. The suppression factor $\eta_{\mathrm{D}}^{(\mathrm{ii})}$ for $I_{\mathrm{DC}}^{\mathrm{D}} = -52\ \mu\mathrm{A}$ is calculated as

$$\eta_{\mathrm{D}}^{(\mathrm{ii})} = \Delta V_{\mathrm{D,S}}^{\mathrm{nl},E\neq 0}\left(I_{\mathrm{DC}}^{\mathrm{D}} = -52\ \mu\mathrm{A}\right)/\Delta V_{\mathrm{S,D}}^{\mathrm{nl}}\left(I_{\mathrm{DC}}^{\mathrm{SD}} = 0\right) = 0.76.$$

With $\eta_{\mathrm{S}}(I = +52\ \mu\mathrm{A}) = \eta_{\mathrm{S}}^{(\mathrm{i})}\eta_{\mathrm{S}}^{(\mathrm{ii})} = 61.8$ and $\eta_{\mathrm{D}}(I = -52\ \mu\mathrm{A}) = \eta_{\mathrm{D}}^{(\mathrm{i})}\eta_{\mathrm{D}}^{(\mathrm{ii})} = 0.34$ we calculate $\Delta V^{E\neq 0}(I = +52\ \mu\mathrm{A}) = \eta_{\mathrm{S}}\Delta V_{\mathrm{S,D}}^{\mathrm{nl}} + \eta_{\mathrm{D}}\Delta V_{\mathrm{D,S}}^{\mathrm{nl}} = 54$ mV, plotted in Fig. 3 as $\Delta R^{E\neq 0}(I = +52\ \mu\mathrm{A}) = \frac{\Delta V^{E\neq 0}}{I} = 1.04\ \mathrm{k}\Omega$ as a red dot. In a similar way, $\Delta R^{E\neq 0}$ values have been obtained for other current values shown in Fig. 3.

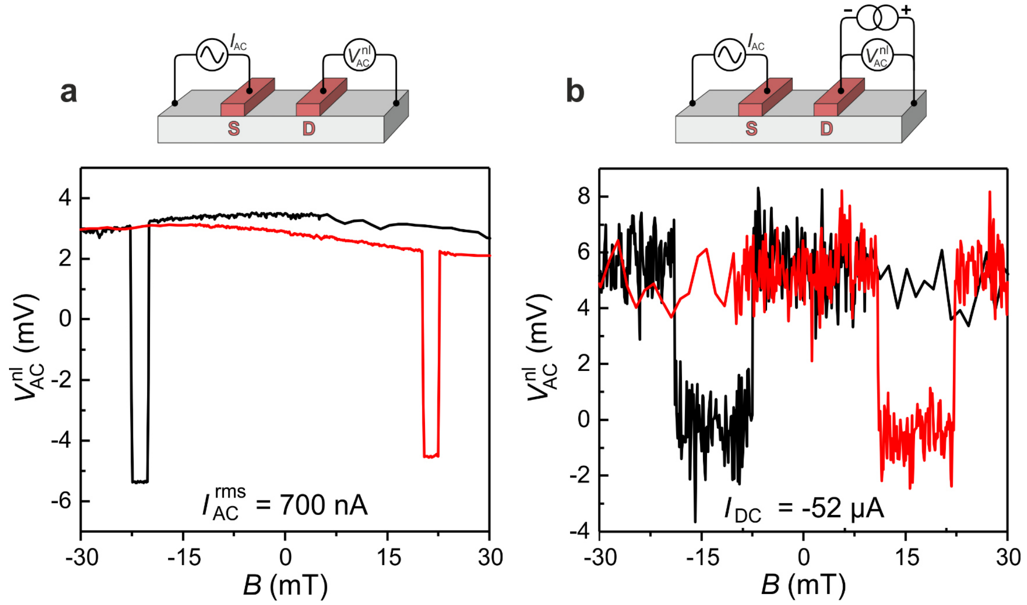

Supplementary Figure 8 | Effect of reverse bias on the nonlocal spin valve signal. Measurements performed in order to determine the suppression of the spin-related voltage detected at the drain, as a result of biasing the lead in the reverse direction. **(a)** Nonlocal spin valve signal detected at the non-biased drain contact with an AC injection current applied to the source. **(b)** The same measurement configuration as in **(a)**, with an additional DC current applied to the nonlocal detector (D).

Supplementary Note 4. Spin diffusion calculation of ΔR in a confined geometry.

In this Note we estimate the effect of confinement on the two-terminal spin resistance in the limit of low electric fields. For simplicity, we assume symmetric tunnel junctions having the same values for spin injection efficiency P and contact resistance R_T. We checked that this assumption does not influence the following conclusions. As $R_\mathrm{F} \ll R_\mathrm{ch} \ll R_\mathrm{T}$ holds for our devices (see Supplementary Note 1), we can estimate the 2T resistance change ΔR_open in the open configuration from the standard diffusion equations:

$$\Delta R_\mathrm{open} = 2P^2 R_\mathrm{ch} \exp\left(-\frac{d}{\lambda_\mathrm{s}}\right) \approx 40\ \Omega \qquad (1)$$

For a confined geometry the formalism of Jaffrès et al.[5] predicts a two-terminal resistance of

$$\Delta R_\mathrm{conf} = \frac{4P^2 R_\mathrm{T}}{2cosh\left(\frac{d}{\lambda_\mathrm{s}}\right) + \left(\frac{R_\mathrm{T}}{R_\mathrm{ch}} + \frac{R_\mathrm{ch}}{R_\mathrm{T}}\right) sinh\left(\frac{d}{\lambda_\mathrm{s}}\right)} \approx 232\ \Omega \qquad (2)$$

for the charge- and spin transport parameters of our device. This corresponds to an enhancement factor of $\Delta R_\mathrm{conf}/\Delta R_\mathrm{open} = 5.8$, which is in good agreement with our low field experimental results (see Fig. 4b).

Supplementary Note 5. Gate control of the magnetoresistance.

In this Note, we show the results of gate-controlled magnetoresistance measurements by repeating the measurement shown in Fig. 4 with another gated device. Supplementary Figure 9a shows *I-V* curves measured between source and drain contacts in both open and confined configurations. Similarly as for the sample discussed in the main text, there is a clear difference of the *I-V* trace for antiparallel magnetization orientation between open and confined configurations. This difference is reflected in the local spin valve measurements, shown in Supplementary Figure 9b, which displays the results for an injection current of I=32 μA. For this current, we observe an increase of the MR ratio from ~3% in the open configuration to ~16% in the confined configuration, corresponding to a total resistance change of ~13%.

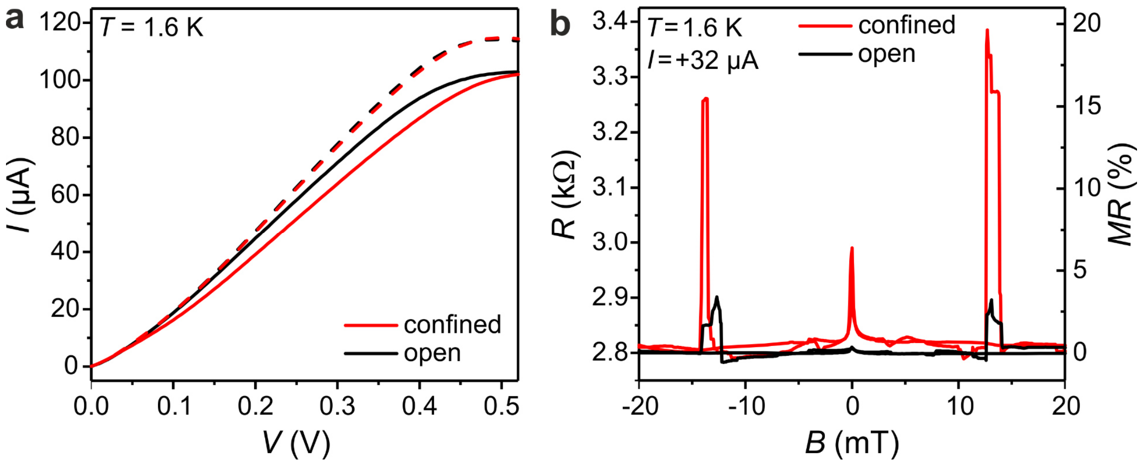

Supplementary Figure 9 | Gate control of the magnetoresistance. (a) *I-V* curve between source and drain contacts in open (black) and confined (red) configuration for parallel (dashed lines) and antiparallel (solid lines) orientation of the magnetization in both leads. **(b)** 2T local spin valve measurements in both open (black) and confined (red) configuration for an injection current *I*=32 μA. Measurements in the open configuration were performed with a gate voltage V_G = +1.5 V and in the confined configuration with V_G = -3 V.

Supplementary Note 6. Confinement by etching.

In this Note, we show the results of additional experiments (Supplementary Figure 10) on samples with the channel confined by etching away parts of the 2DES by reactive ion etching (RIE). Confinement was realized by etching two 3 µm wide trenches to the left and to the right of the corresponding ferromagnetic contacts (see the left bottom picture in Supplementary Figure 10). Both trenches are ~240 nm deep, counting from the top of the mesa. This corresponds to etching away material down to the δ-doped layer of the used wafer (see Supplementary Figure 1a and Methods). We compare the results of two-terminal (2T) spin valve (SV) measurements performed on the etch-confined device with the non-etched devices, i.e., in the open geometry. Both devices were fabricated on the same piece of wafer and the measurements were performed during the same cooldown. We observe consistent results with those obtained from gated samples, i.e., increase of the MR with confinement and the effect of confinement reduced for higher bias currents.

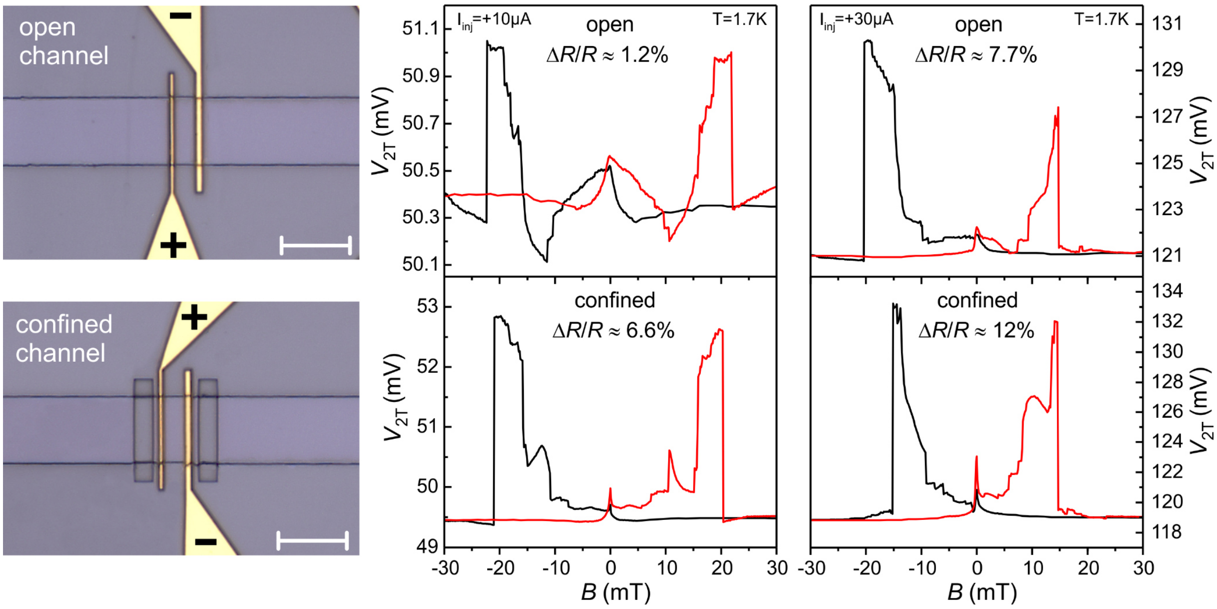

Supplementary Figure 10 | Confinement by physical etching. Spin valve measurements in the confined and in the open configuration with the confinement realized by RIE etching. Left panel: Microscope pictures of the open and confined devices. RIE-etched trenches are visible at the bottom picture to the left and to the right of corresponding ferromagnetic contacts. Right panel: 2T SV measurements for I=10 µA and 30 µA. Polarity of the applied voltage is shown in the left panel. Scale bars correspond to 10 µm.

Supplementary Note 7. Two-terminal local Hanle measurements.

In this Note we summarize the results of two-terminal Hanle measurements, i.e., measurements in an external out-of-plane magnetic field. Suppression of the measured signal in the field direction transverse to the injected spins, with the amplitude of the signal fully consistent with local spin valve signals, confirms the spin origin of the measured magnetoresistance. As we reported in our recent work on nonlocal Hanle experiments in 2DES samples[19], Hanle curves are strongly affected by dynamic nuclear polarization (DNP) effects[20], which lead to narrowing of the curves through the additional effective magnetic field acting on electron spin. These effects are particularly strong for large bias voltages, when large spin accumulation is induced in the channel. This makes quantitative analysis of the experimental data, particularly extraction of the spin relaxation times, very difficult. As we showed in Ref. 19, one can limit the influence of the DNP by performing AC low excitation measurements. We show the example of such a local Hanle curve in Supplementary Figure 11b. Although these measured curves are clearly wider than for a DC current of I_{inj}=–100 µA, DNP effects are still visible in the data for the AP configuration, e.g., through a shoulder for negative values of B.

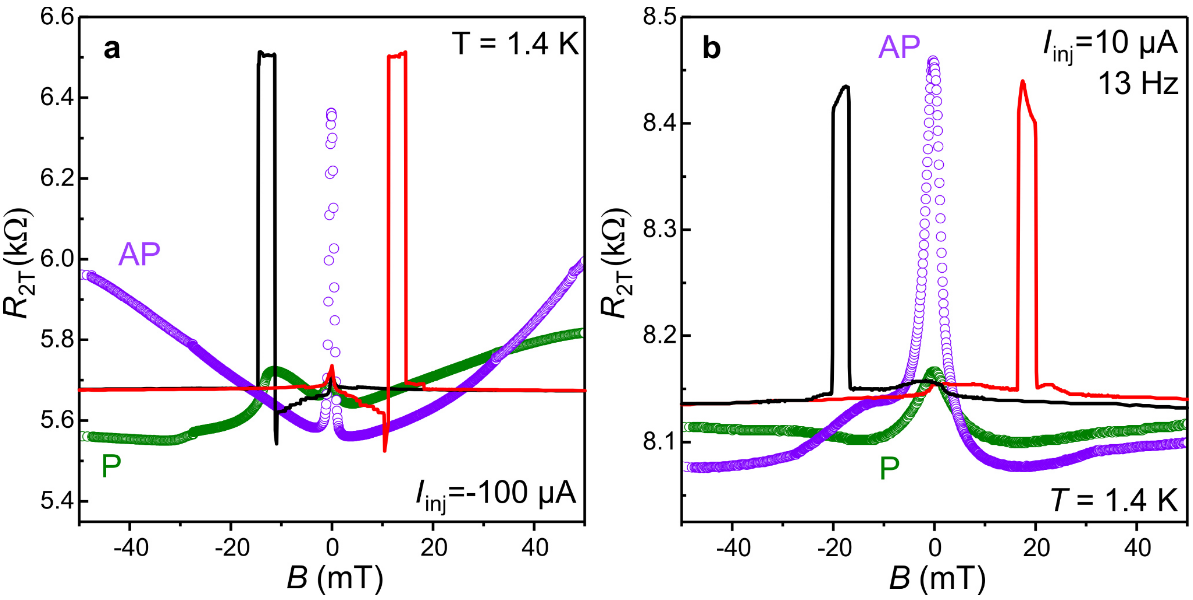

Supplementary Figure 11 | Hanle depolarization experiments. Two-terminal local Hanle measurements for **(a)** DC injection current of I_{inj}=-100 µA and **(b)** AC injection current I_{inj}=10 µA with frequency f=13 Hz. Measurements performed for both parallel (P) and antiparallel (AP) orientation of source and drain contacts. Spin valve traces (red and black lines) are shown for comparison. Measurement were performed on the same device as presented in Fig. 2.

Supplementary Note 8. Temperature dependence of the local spin valve signal.

In this Note we show two-terminal local spin valve signals measured at different temperatures. The amplitude of the signal drops with temperature as we approach the Curie temperature of used (Ga,Mn)As layer, T_c=55 K. The SV pattern moves for higher temperatures towards B=0 as the coercive fields of the magnetic contacts decrease.

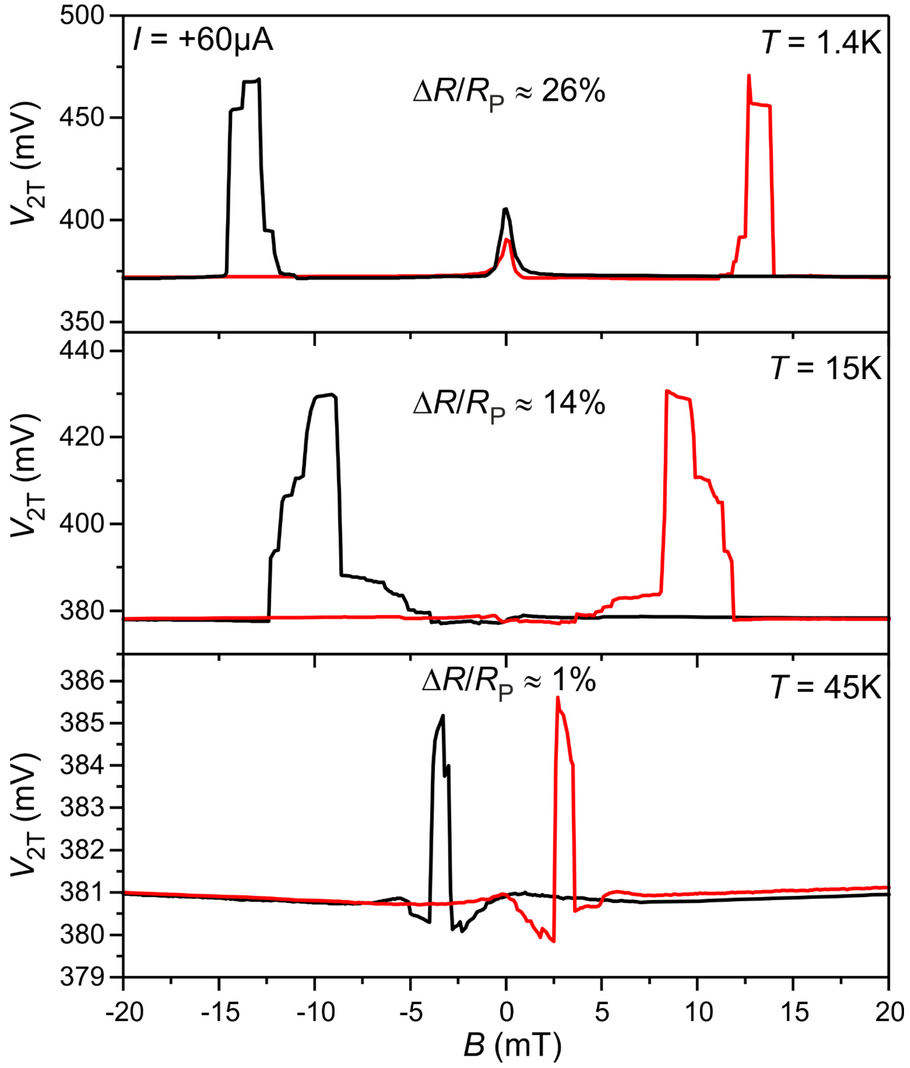

Supplementary Figure 12 | Temperature dependence of the 2T spin valve signal Two-terminal local spin valve signal for a DC injection current of I_{inj}=+60 μA at different temperatures. Measurement performed on the same device as presented in Fig. 2.

References

1. Oltscher, M. et al. Electrical Spin Injection into High Mobility 2D Systems. *Phys. Rev. Lett.* **113,** 236602 (2014).
2. Ciorga, M. Electrical spin injection and detection in high mobility 2DEG systems. *J. Phys. Condens. Matter* **28,** 453003 (2016).
3. Johnson, M. & Silsbee, R. H. Coupling of electronic charge and spin at a ferromagnetic-paramagnetic metal interface. *Phys. Rev. B* **37,** 5312–5325 (1988).
4. Fabian, J. & Zutic, I. The standard model of spin injection. in *Spintronics- from GMR to Quantum Information* C1 (2009).
5. Jaffrès, H., George, J. M. & Fert, A. Spin transport in multiterminal devices: Large spin signals in devices with confined geometry. *Phys. Rev. B* **82,** 140408 (2010).
6. Fert, A., George, J.-M., Jaffres, H. & Mattana, R. Semiconductors Between Spin-Polarized Sources and Drains. *IEEE Trans. Electron Devices* **54,** 921–932 (2007).
7. Hueso, L. E. et al. Transformation of spin information into large electrical signals using carbon nanotubes. *Nature* **445,** 410–413 (2007).
8. Bruski, P. et al. All-electrical spin injection and detection in the Co2FeSi/GaAs hybrid system in the local and non-local configuration. *Appl. Phys. Lett.* **103,** 52406 (2013).
9. Sasaki, T. et al. Local magnetoresistance in Fe/MgO/Si lateral spin valve at room temperature. *Appl. Phys. Lett.* **104,** 52404 (2014).
10. Tahara, T. et al. Room-temperature operation of Si spin MOSFET with high on/off spin signal ratio. *Appl. Phys. Express* **8,** 11–14 (2015).
11. Ciorga, M. et al. Local spin valve effect in lateral (Ga,Mn)As/GaAs spin Esaki diode devices. *AIP Adv.* **1,** 22113 (2011).
12. Koo, H. C. et al. Electrical spin injection and detection in an InAs quantum well. *Appl. Phys. Lett.* **90,** 22101 (2007).
13. Nakane, R., Harada, T., Sugiura, K. & Tanaka, M. Magnetoresistance of a spin metal-oxide-semiconductor field-effect transistor with ferromagnetic MnAs source and drain contacts. *Jpn. J. Appl. Phys.* **49,** (2010).
14. Yu, Z. & Flatté, M. Spin diffusion and injection in semiconductor structures: Electric field effects. *Phys. Rev. B* **66,** 235302 (2002).
15. Chantis, A. N. & Smith, D. L. Theory of electrical spin-detection at a ferromagnet/semiconductor interface. *Phys. Rev. B* **78,** 235317 (2008).
16. Crooker, S. a. et al. Bias-controlled sensitivity of ferromagnet/semiconductor electrical spin detectors. *Phys. Rev. B* **80,** 41305 (2009).
17. Tahara, T. et al. Observation of large spin accumulation voltages in nondegenerate Si spin devices due to spin drift effect: Experiments and theory. *Phys. Rev. B* **93,** 214406 (2016).
18. Buchner, M. et al. Optical investigation of electrical spin injection into an inverted two-dimensional electron gas structure. *Phys. Rev. B* **95,** 35304 (2017).
19. Kuczmik, T. et al. Hanle spin precession in a two-dimensional electron system. *Phys. Rev. B* **95,** 195315 (2017)

20. Salis, G., Fuhrer, A. & Alvarado, S. F. Signatures of dynamically polarized nuclear spins in all-electrical lateral spin transport devices. *Phys. Rev. B* **80,** 115332 (2009).
